# Changes in patient subjective happiness and satisfaction with cataract surgery

**DOI:** 10.1038/s41598-020-72846-2

**Published:** 2020-10-14

**Authors:** Erisa Yotsukura, Masahiko Ayaki, Naohisa Nezu, Hidemasa Torii, Hiroyuki Arai, Keiko Sakatani, Kazuo Tsubota, Kazuno Negishi

**Affiliations:** 1 Department of Ophthalmology, Keio University School of Medicine, 35 Shinanomachi, Shinjuku-ku, Tokyo 160-8582 Japan; 2 Otake Clinic Moon View Eye Center, 521-8 Shimotsuruma, Yamato City, Kanagawa 242-0001 Japan; 3 Todoroki Eye Clinic, 3-10-12 Todoroki, Setagaya-ku, Tokyo 158-0008 Japan; 4 Minatomirai Eye Clinic, 2-3-5 Minatomirai, Nishi-ku, Yokohama-shi, Kanagawa 220-6208 Japan

**Keywords:** Quality of life, Lens diseases

## Abstract

The purpose of this study was to investigate the changes in patient subjective happiness and satisfaction with cataract surgery and evaluate the association between satisfaction and types of cataract. This study surveyed 247 participants (mean age, 67.9 years) and they completed questionnaires on their satisfaction with the surgery, the subjective happiness scale (SHS) and the Pittsburgh Sleep Quality Index (PSQI) before and after surgery. The SHS increased postoperatively from 4.6 ± 0.7 to 4.8 ± 0.7 (*P* = 0.007) and 83.4% of patients were satisfied with the surgical results and the average satisfaction score was 4.2 out of a possible 5.0. Multiple regression analysis showed that patient satisfaction was significantly associated with the postoperative SHS (β = 0.380; *P* < 0.001), the postoperative PSQI (β = −0.041; *P* = 0.035) and the presence of a posterior subcapsular cataract (PSC) (β = 0.277; *P* = 0.026). This study clarified that cataract surgery may improve both visual function and patient happiness and that patient satisfaction was affected by postoperative sleep quality and the disappearance of a PSC.

Cataracts are common in elderly individuals, and surgery is indicated in most cases. Several studies have reported that cataract surgery improves not only the visual function but also the patients’ quality of life (QOL). Previous studies have reported that the risk of falls decreased after cataract surgery^1–3^ and that cataract surgery can improve postural stability and mobility^4,5^. Another study reported improvements in the QOL, cognition, and depression after cataract surgery^6^. We evaluated changes in the sleep quality and gait speed before and after cataract surgery and reported that surgery effectively improved sleep quality and slow gaid speed^7^. Therefore, cataract surgery can improve the patients’ lives.

Recent psychology research has indicated that happiness has become an important goal for many people^8^, and positive emotions are highly beneficial for physical and mental health^9–13^. In Ophthalmology, Kawashima et al. reported an association between subjective happiness and dry eye disease^14^. Matsuguma et al. evaluated the relationship between subjective happiness and laser-assisted in situ keratomileusis^15^ and visual impairment^16^. A recent review reported that positive affective states, including happiness, may contribute to sleep^17^. As mentioned previously, using the Pittsburgh Sleep Quality Index (PSQI), we found that cataract surgery effectively improved sleep quality and that the PSQI improved significantly in patients with a posterior subcapsular cataract (PSC)^18^. Considering these findings, we assumed that the patient subjective happiness and sleep quality also may be better postoperatively. Feeny et al. showed that in Vietnam cataract surgery provided benefits that included significant improvements in earnings, mobility, self-care, ability to undertake daily activities, self-assessed health and mental health, life satisfaction, hope, and self-efficacy^19^.

In the current study, we investigated the changes in the patient subjective happiness before and after cataract surgery and surgical satisfaction in Japan and the association between subjective happiness, satisfaction, and cataract-related factors by evaluating the types and severity of cataracts.

## Results

### Results of ocular examinations and questionnaires

During the study, 261 patients underwent cataract surgery and 14 were excluded. The 247 remaining patients (99 men, 148 women; mean age, 67.9 ± 11.4 years; range, 32–92 years) were evaluated. Seventy-four patients underwent unilateral surgery and 173 underwent bilateral surgeries. Fifty-six patients of undergoing unilateral surgery were the first-eye surgery and 18 patients were second-eye surgery. There were not any complications including posterior capsule rupture.

The preoperative and postoperative results of ocular examinations and questionnaires are summarized in Table 1. The postoperative SHS increased significantly (*P* = 0.007) from 4.6 ± 0.7 to 4.8 ± 0.7. The average satisfaction score was 4.2 ± 0.9; 83.4% of patients were satisfied with the surgery (very satisfied, n = 108; satisfied, n = 98). The postoperative CDVA, UDVA, and PSQI were significantly better than the preoperative values (CDVA and UDVA, *P* < 0.001 for both comparisons; PSQI, *P* = 0.009).

**Table 1:** Results of ocular examinations and questionnaires.

Variable	Preoperative Mean ± standard deviation [SD] (range)	Postoperative Mean ± SD (range)	*P* value
Corrected distance visual acuity (logMAR)	0.28 ± 0.52 (− 1.18 to 4.00)	− 0.16 ± 0.21 (− 1.10 to 0.80)	< .001
Uncorrected distance visual acuity (logMAR)	0.79 ± 0.59 (− 0.079 to 4.00)	0.51 ± 0.55 (− 0.18 to 3.0)	< .001
Refractive error (diopters)	− 2.14 ± 4.64 (− 21.0 to 5.5)	− 0.57 ± 0.98 (− 6.0 to 1.0)	< .001
SHS score	4.6 ± 0.7 (2.5–7.0)	4.8 ± 0.7 (3.0–7.0)	.007
PSQI score	5.1 ± 2.7 (3–10)	4.8 ± 2.7 (0–15)	.009
Sleep time	6.7 ± 1.1 (0–14)	6.7 ± 1.0 (3.0–10.0)	.462
Satisfaction score	–	4.2 ± 0.9 (1–5)	–

*SD* standard deviation, *logMAR* logarithm of the minimum angle of resolution, *SHS* Subjective Happiness Scale, *PSQI* Pittsburgh Sleep Quality Index.

There are not significant differences between first-eye surgery group and second-eye surgery group in unilateral surgery patients about satisfaction score, change in SHS and PSQI (Table 2).

**Table 2:** Comparison of variables in unilateral patients.

Variable	Total (n = 74) Mean ± SD	First surgery (n = 56) Mean ± SD	Second surgery (n = 18) Mean ± SD	*P* value
Age	62.4 ± 13.4	61.1 ± 13.2	66.3 ± 13.4	.083
Sex	0.5 ± 0.5	0.5 ± 0.5	0.5 ± 0.5	.896
Preoperative SHS	4.7 ± 0.7	4.7 ± 0.7	4.7 ± 0.7	.914
Preoperative PSQI	5.4 ± 2.9	5.8 ± 3.1	4.2 ± 1.8	.092
Postoperative SHS	4.7 ± 0.7	4.7 ± 0.7	4.8 ± 0.6	.417
Postoperative PSQI	4.9 ± 2.7	5.3 ± 2.8	3.8 ± 2.1	.070
Satisfaction score	4.3 ± 0.8	4.4 ± 0.7	4.2 ± 1.8	.517
Change in CDVA	− 0.67 ± 0.78	− 0.75 ± 0.84	− 0.42 ± 0.47	.064
Change in SHS	0.1 ± 0.7	0.04 ± 0.8	0.1 ± 0.5	.809
Change in PSQI	− 0.5 ± 2.0	− 0.5 ± 2.2	− 0.4 ± 1.3	.802

Sex: Male = 0, Female = 1; *SHS* Subjective Happiness Scale, *PSQI* Pittsburgh Sleep Quality Index, *CDVA* corrected distance visual acuity.

The numbers of patients with nuclear opalescence (≥ grade 3), cortical cataract, and PSC were 54, 71, and 54, respectively. The presence of nuclear opalescence was significantly (*P* = 0.035) more prevalent in women than men (*P* = 0.035), and their refractive error was significantly (*P* < 0.01) more myopic than the patients without nuclear opalescence (− 4.79 ± 6.11 diopters and − 1.37 ± 3.80 diopters, respectively).

### Factors associated with subjective happiness

Table 3 shows the factors associated with the preoperative SHS results. The presence of nuclear opalescence (≥ grade 3) (β = 0.276; *P* = 0.017) and the preoperative CDVA (β = −0.248; *P* = 0.008) significantly affected the patient happiness before cataract surgery. Table 4 shows the factors associated with the postoperative SHS results, then the postoperative SHS was associated significantly (β = 0.334; *P* < 0.001) with the preoperative SHS. The satisfaction score was significantly (β = 0.169; *P* = 0.005) associated with the changes in the SHS (Table 5).

**Table 3:** Factors associated with subjective happiness preoperatively.

Variable	Univariate estimate	Multivariate estimate		
	*P* value	β	95% CI	*P* value
Age	.032*			.144
Sex	.409			
No. operated eyes	.296			
Nuclear opalescence (≥ grade 3)	.030*	0.276	0.050–0.503	.017
Cortical cataract	.075*			.762
Posterior subcapsular cataract	.455			
Preoperative CDVA	.014*	− 0.248	− 0.43 to − 0.066	.008
Preoperative SE	.463			
Preoperative PSQI	.073*			.122

Sex: Male = 0, Female = 1; *CI* confidence interval, *CDVA* corrected distance visual acuity, *SE* spherical equivalent, *SHS* Subjective Happiness Scale.

*Variables which *P* values were less than 0.20 in simple correlation were performed with multiple regression analysis.

**Table 4:** Factors Associated with the Subjective Happiness Postoperatively.

Variable	Univariate estimate	Multivariate estimate		
	*P* value	β	95% CI	*P* value
Age	.001*	0.010	0.002–0.017	.010
Sex	.056*			.199
No. operated eyes	.171*			.732
Nuclear opalescence (≥ grade 3)	.340			
Cortical cataract	.163*			.977
Posterior subcapsular cataract	.286			
Preoperative SHS	< .001*	0.334	0.222–0.446	< .001
Preoperative PSQI	.059*			.358
Postoperative CDVA	.170*			.225
Postoperative UDVA	.174*			.571
Postoperative SE	.191*			.663

Sex: Male = 0, Female = 1; *CI* confidence interval, *SHS* Subjective Happiness Scale, *PSQI* Pittsburgh Sleep Quality Index, *CDVA* corrected distance visual acuity, *UDVA* uncorrected distance visual acuity, *SE* spherical equivalent.

*Variables which *P* values were less than 0.20 in simple correlation were performed with multiple regression analysis.

**Table 5:** Factors associated with changes in subjective happiness.

Variable	Univariate estimate	Multivariate estimate		
	*P* value	β	95% CI	*P* value
Age	.150*			.341
Sex	.100*			.201
No. operated eyes	.148*			.184
Nuclear opalescence (≥ grade 3)	.109*			.111
Cortical cataract	.249			
Posterior subcapsular cataract	.168*			.623
Change in CDVA	.319			
Change in PSQI	.116*			.386
Satisfaction score	.003*	0.169	0.051–0.287	.005

Sex: Male = 0; Female = 1; *CI* confidence interval, *CDVA* corrected distance visual acuity, *PSQI* Pittsburgh Sleep Quality Index.

*Variables which *P* values were less than 0.20 in simple correlation were performed with multiple regression analysis.

### Factors affecting surgical satisfaction

**Figure 1:**
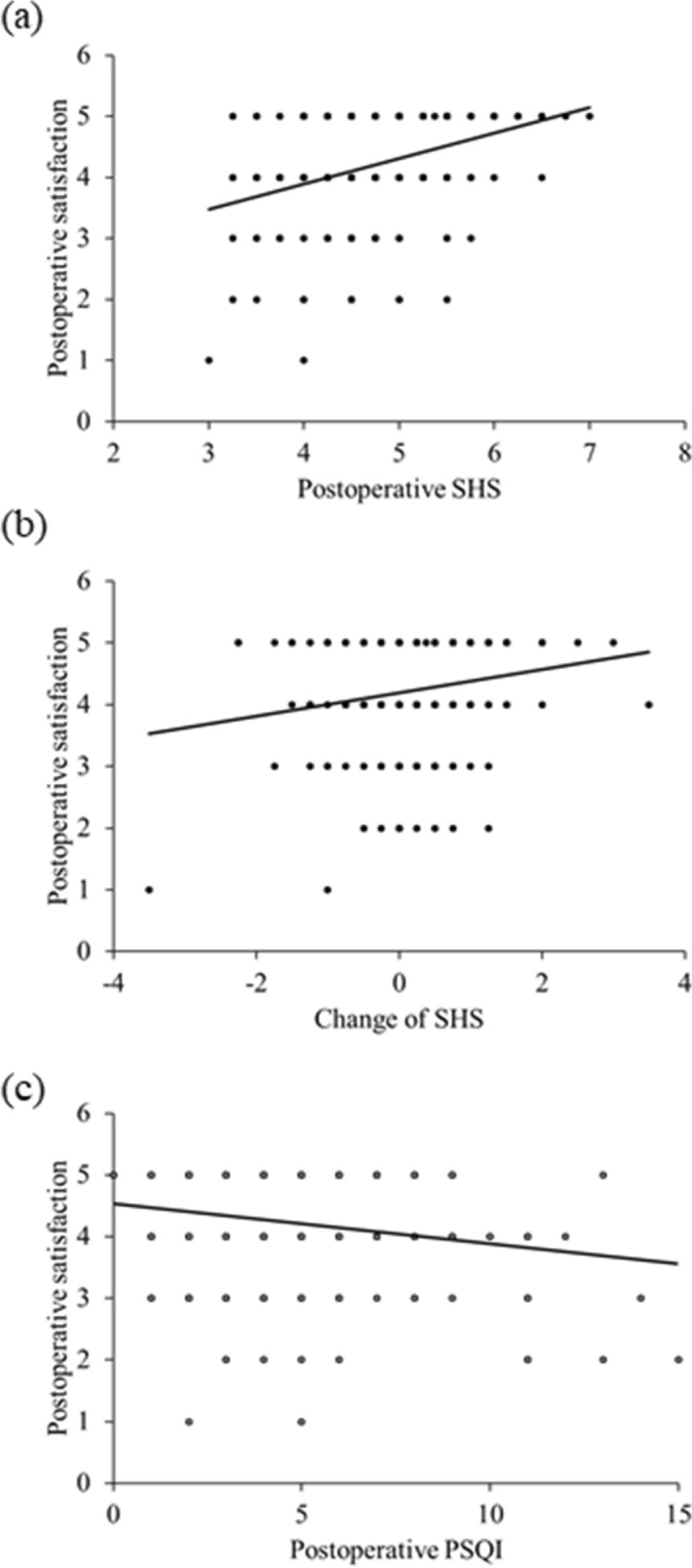
The Correlation Between Patient Satisfaction and Postoperative Subjective Happiness Scale (SHS)/Changes in SHS/Pittsburgh Sleep Quality Index (PSQI). (**a**) The patient satisfaction is correlated significantly with the postoperative SHS score (r = 0.324, *P* < 0.001). (**b**) The patient satisfaction is correlated significantly with the changes in the SHS score (r = 0.157, *P* = 0.013). (**c**) The patient satisfaction is correlated significantly with the postoperative PSQI score (r = −0.144, *P* = 0.024).

The correlations between patient satisfaction and the postoperative SHS, changes in the SHS, and postoperative PSQI are shown in Fig. 1 respectively. Patient satisfaction was correlated significantly with the postoperative SHS (r = 0.324, *P* < 0.001) (Fig. 1a), changes in the SHS (r = 0.157, *P* = 0.013) (Fig. 1b) and postoperative PSQI (r = −0.144, *P* = 0.024) (Fig. 1c).

**Figure 2:**
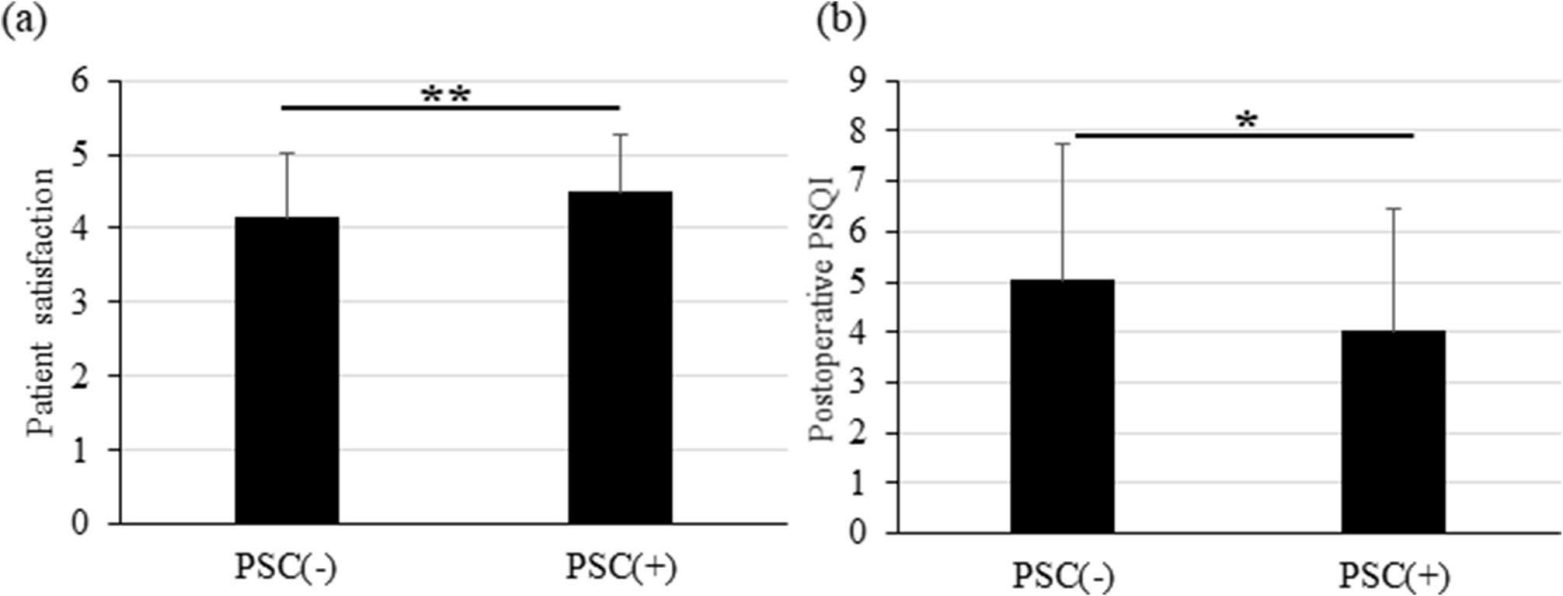
Patient Satisfaction and Postoperative Pittsburgh Sleep Quality Index (PSQI) Scores with and without a Posterior Subcapsular Cataract (PSC). (**a**) The patient satisfaction scores with and without a PSC are 4.50 ± 0.76 and 4.14 ± 0.86, respectively, and the mean score of patients with a PSC is significantly (*P* = 0.004) higher than without a PSC. (**b**) The postoperative PSQI scores with and without a PSC are 4.02 ± 2.44 and 5.04 ± 2.70, respectively. The score of patients with a PSC is significantly (*P* = 0.010) better than without a PSC.

Multiple regression analysis showed that the postoperative SHS (β = 0.380; *P* < 0.001), the postoperative PSQI (β = −0.041; *P* = 0.035) and the presence of a PSC (β = 0.277; *P* = 0.026) significantly affected the patient surgical satisfaction (Table 6). The patient satisfaction scores with or without a PSC were 4.50 ± 0.76 and 4.14 ± 0.86, respectively, and the score of patients with a PSC was higher significantly (*P* = 0.004) than without a PSC (Fig. 2a). The postoperative PSQI scores with and without a PSC were 4.02 ± 2.44 and 5.04 ± 2.70, respectively (*P* = 0.010) (Fig. 2b).

**Table 6:** Factors associated with the Satisfaction Score.

Variable	Univariate estimate	Multivariate estimate		
	*P* value	β	95% CI	*P* value
Age	.287			
Sex	.456			
No. operated eyes	.072*			.107
Nuclear opalescence (≥ grade 3)	.034*			.088
Cortical cataract	.006*			.155
Posterior subcapsular cataract	.003*	0.277	0.033–0.520	.026
Postoperative CDVA	.146*			.516
Postoperative UDVA	.393			
Postoperative SHS	< .001*	0.380	0.236–0.523	< .001
Postoperative PSQI	.001*	− 0.041	− 0.079 to − 0.003	.035

Sex: male = 0, female = 1; *CDVA* corrected distance visual acuity, *UDVA* uncorrected distance visual acuity, *SHS* Subjective Happiness Scale, *PSQI* Pittsburgh Sleep Quality Index.

*Variables which *P* values were less than 0.20 in simple correlation were performed with multiple regression analysis.

## Discussion

The current study was the first to investigate the effect of cataract surgery on patient subjective happiness and the relationship between patient surgical satisfaction and subjective happiness. We found that cataract surgery improved both the visual function and the patient subjective happiness postoperatively.

Using the SHS, Kawashima et al.^14^ evaluated the subjective happiness of office workers with subjective and/or objective dry eye symptoms. That study reported that the mean SHS score among patients with subjective and objective dry eye symptoms was 4.87 ± 1.00. A previous study reported that the mean SHS in a sample of healthy Japanese college students was 4.68 ± 1.05^20^. In the current study, the preoperative SHS score of patients with cataract was 4.64 ± 0.73; nevertheless, Shimai et al. reported that the SHS score had been increasing continuously in patients over 50 years old in Japan and the mean SHS score among 60- to 65-year-olds was 4.83 ± 0.99^21^. For these reasons, the results of the current study indicated that declines in visual function resulting from the presence of cataracts have a negative effect on patient happiness.

In the current study, the disappearance of a PSC and improved sleep quality were associated with the patient surgical satisfaction. We previously reported correlations between subjective sleep quality and cataract surgery, especially that patients with a PSC achieved the greatest improvement in subjective sleep quality measured using the PSQI^18^. Cataracts increase the intraocular light scattering and thereby reduce the retinal image contrast^22,23^. Previous studies have reported that PSC especially causes reduced contrast sensitivity^23,24^ and objective glare disability is more commonly associated with PSC than with other cataract types^25^. In addition, patients with a PSC had considerably impaired visual function and the self-reported visual satisfaction was significantly lower than with the other cataract types^26^. Therefore, it is reasonable to speculate that patients with a PSC had impaired vision with reduced contrast sensitivity and glare disability and had great surgical satisfaction because their preoperative discomfort improved after cataract surgery.

Many previous studies have evaluated sleep quality before and after cataract surgery; two meta-analyses^27,28^ showed that cataract surgery significantly improved the PSQI score-derived subjective sleep quality irrespective of the intraocular lens (IOL) type implanted. The current study confirmed that sleep quality improved significantly after cataract surgery and further confirmed the association between the improvement and patient satisfaction.

Interestingly, we also found that the preoperative SHS score was associated with poor VA and the presence of nuclear opalescence. The patients with poor VA had a lower SHS score, and those with nuclear opalescence had a higher SHS score. More women than men were among those with nuclear opalescence. Shimai et al. reported that the SHS scores of women were higher than those of men^20,21^. The current results agree with those of previous studies. In addition, refractive error of the patients with nuclear opalescence in this study was more myopic than the patients without nuclear opalescence. We speculated that better near vision in myopic patients with nuclear opalescence may account for their higher SHS score compared with patients without nuclear opalescence.

The current study had some limitations. First, we could not collect additional data that might have affected subjective happiness, such as income or marital status. We inferred that the patients with nuclear opalescence may have elements that made them happier than those without nuclear opalescence. Second, we did not evaluate the improvement of symptoms such as glare or haloes. We should have assessed these points with questionnaire, glare testing and low contrast visual acuity testing, because these factors possibly affected the patient’s daily life. Third, the study included some biases because it was conducted in one hospital and two clinics in an urban area. Fourth, the patients’ happiness may change over time, considering the set-point theory of happiness^29^. We should follow these patients for longer periods, such as 6 months or 1 year. Finally, this study was conducted in a mixed sample that included two types of IOLs (a clear ultraviolet light-filtering IOL and a yellow blue light-filtering IOL) and unilateral/bilateral cataracts.

## Methods

### Study design and study population

The study participants were patients who underwent cataract surgery at Todoroki Eye Clinic, Tokyo, Japan, Minatomirai Eye Clinic, Kanagawa, Japan, and Keio University Hospital, Tokyo, Japan, between June 2016 and June 2018. The subjects completed questionnaires that measured their subjective happiness within 1 month before and after surgery and their satisfaction 1 month with surgery.

Patients were excluded who were younger than 20 years, did not answer all questions, and Took a psychotropic drug. The Institutional Review Board of Keio University School of Medicine approved the research protocol, and the study was conducted in accordance with the tenets of the 1995 Declaration of Helsinki. All participants were provided informed consent.

### Ocular examinations

All participants underwent preoperative and postoperative ocular examinations that included measurement of the uncorrected distance visual acuity (UDVA), corrected distance VA (CDVA), and spherical equivalent (SE) by an orthoptist. The SE is a commonly used to express the refractive error by algebraically adding half the cylindrical power to the spherical power. Three experienced ophthalmologists who performed the cataract surgeries evaluated the types and severity of the cataracts by slit-lamp biomicroscopy under maximal pupillary dilation. The types of cataracts were classified according to the Lens Opacities Classification System III^30^ as nuclear opalescence grade 3 or higher, cortical cataracts disrupting the visual axis, and PSCs. Nuclear opalescence was graded by comparing the color slit-lamp image to be graded with the standard nuclear images (standards 1–6)^30^. A cortical cataract is visualized in retroillumination images focused either anteriorly or posteriorly^30^. Only posteriorly focused retroillumination images are used in grading PSC^30^.

### Surgical technique

All surgeons underwent phacoemulsification and aspiration. The selection of anesthesia during surgery, two of them underwent with only topical anesthesia (oxybuprocaine hydrochloride and lidocaine hydrochloride 4.0%) and another clinic underwent both topical anesthesia and intracameral anesthesia (lidocaine hydrochloride 0.5%). After the surgery, two medical institutions prescribed three ophthalmic solutions (levofloxacin hydrate 1.5%, betamethasone sodium phosphate 0.1% and bromfenac sodium hydrate 0.1% or moxifloxacin hydrochloride 0.5%, betamethasone sodium phosphate 0.1% and diclofenac sodium ophthalmic solution 0.1%) for postoperative medication. Another clinic prescribed two ophthalmic solutions (moxifloxacin hydrochloride 0.5% and bromfenac sodium hydrate 0.1%) and add a corticosteroid ophthalmic solution (betamethasone sodium phosphate fradiomycin sulfate) in case the patient has severe inflammation after the surgery.

### Subjective happiness scale

Regarding the outcome measures, subjective happiness was measured using the validated Japanese version of the Subjective Happiness Scale (SHS)^20^ originally developed by Lyubomirsky (eTable 1)^31^. The scale is a four-item questionnaire of subjective global happiness; each item requires patients to rate the statements on a 7-point Likert scale. The possible scores range from 1 to 7, and higher values corresponded to higher subjective happiness. The overall SHS score was calculated by calculating the mean of the responses to the four items. Both the internal consistency (α = 0.80 for men; α = 0.84 for women) and test–retest reliability (r = 0.86) over 5 weeks were scientifically sound^31^.

### Satisfaction score

We also evaluated the patient postoperative satisfaction using the original questionnaire. The questionnaire used a 5-point Likert scale ranging from 1 (least satisfied) to 5 (very satisfied).

### Pittsburgh sleep quality index

The PSQI^32^, a self-administered questionnaire, is comprised of seven subscales that evaluate sleep quality, including subjective sleep quality, daytime dysfunction, sleep latency, sleep duration, habitual sleep efficacy, sleep disturbances, and use of sleep medications; the score was calculated according to separate algorithms and analyzed. Each component was scored on a scale of 0–3, with 3 indicating the worst score. The highest possible global score is 21, and the normal range on the PSQI is less than 6^32^. We used the validated Japanese version of the PSQI^33^.

### Statistical analysis

If the patient underwent bilateral surgeries, the data from the eye with the better VA were used for all statistical analyses. The VA was converted to the logarithm of the minimum angle of resolution (logMAR). For the statistical analysis of the CDVA and UDCA, counting fingers was categorized as logMAR 2.00 and hand motions as logMAR 3.00, according to the VA conversion chart of the *Journal of Cataract & Refractive Surgery*.

The differences between the preoperative and postoperative CDVA, UDVA, SE, SHS, and PSQI were analyzed using the Wilcoxon signed-rank test. The differences between first-eye surgery group and second-eye surgery group in unilateral surgery patients were also analyzed using the Wilcoxon signed-rank test.

The correlations were evaluated using the Spearman’s rank correlation test. Multiple regression analysis was performed for potential variables with statistical significance (*P* < 0.20) in simple correlation to identify the factors affecting the patient satisfaction and happiness with the results of cataract surgery. Multiple regression analysis then was performed to determine the predictors of the SHS score and satisfaction, and possible predictors (sex; age; number of operated eyes; presence of nuclear opalescence, cortical cataract and PSC; and preoperative or postoperative CDVA, UDVA, SHS, and PSQI). *P* < 0.05 was considered significant. All statistical analyses were performed using SPSS version 25 (SPSS Inc., Chicago, IL).

## Conclusions

In summary, the current study showed that cataract surgery may improve not only the visual function but also the patient happiness with surgery. The study also showed that the patient postoperative subjective happiness was associated with surgical satisfaction, and the satisfaction was affected by postoperative sleep quality and the disappearance of a PSC.

## Supplementary information

Supplementary Table.
