# Supplementary material for: Changes in patient subjective happiness and satisfaction with cataract surgery
Source: Sci Rep. 2020 Oct 14;10:17273. doi: 10.1038/s41598-020-72846-2 (PMC7560890; doi:10.1038/s41598-020-72846-2)
Supplement: Supplementary file 1 — Supplementary Table. [file 41598_2020_72846_MOESM1_ESM.docx]

**Supplement**

**eTable 1. The Four Questions included in the Subjective Happiness Scale.**

| 1. In general, I consider myself  (not a very happy person) 1 2 3 4 5 6 7 (a very happy person). |
| --- |
| 2. Compared with most of my peers, I consider myself  (less happy) 1 2 3 4 5 6 7 (happier). |
| 3. Some people are generally very happy. They enjoy life regardless of what is going on, getting the most out of everything. To what extent does this characterization describe you?  (not at all) 1 2 3 4 5 6 7 (a great deal) |
| 4. Some people are generally not very happy. Although they are not depressed, they never seem as happy as they might be. To what extent does this characterization describe you?  (not at all) 1 2 3 4 5 6 7 (a great deal)* |
|  |
